# Matching the Cellulose/Silica Films Surface Properties for Design of Biomaterials That Modulate Extracellular Matrix

**DOI:** 10.3390/membranes11110840

**Published:** 2021-10-29

**Authors:** Adina-Maria Dobos, Elena-Laura Ursu, Luiza-Madalina Gradinaru, Marius Dobromir, Anca Filimon

**Affiliations:** 1 Polycondensation and Thermostable Polymers Department, “Petru Poni” Institute of Macromolecular Chemistry, Grigore Ghica Voda Alley No. 41A, 700506 Iasi, Romania; 2 Centre of Advanced Research in Bionanoconjugates and Biopolymers Department, “Petru Poni” Institute of Macromolecular Chemistry, Grigore Ghica Voda Alley No. 41A, 700506 Iasi, Romania; ursu.laura@icmpp.ro; 3 Polyaddition and Photochemistry Laboratory, “Petru Poni” Institute of Macromolecular Chemistry, Grigore Ghica Voda Alley No. 41A, 700506 Iasi, Romania; gradinaru.luiza@icmpp.ro; 4 Department of Exact and Natural Sciences, Institute of Interdisciplinary Research, “Alexandru Ioan Cuza” University, 11 Carol I Blvd., 700506 Iasi, Romania; marius.dobromir@uaic.ro

**Keywords:** cellulose acetate/silica composite films, surface morphology, wettability properties, biointerface interaction

## Abstract

The surface properties of composite films are important to know for many applications from the industrial domain to the medical domain. The physical and chemical characteristics of film/membrane surfaces are totally different from those of the bulk due to the surface segregation of the low surface energy components. Thus, the surfaces of cellulose acetate/silica composite films are analyzed in order to obtain information on the morphology, topography and wettability through atomic force microscopy (AFM), X-ray photoelectron spectroscopy (XPS) and contact angle investigations. The studied composite films present different surface properties depending on the tetraethyl orthosilicate (TEOS) content from the casting solutions. Up to a content of 1.5 wt.% TEOS, the surface roughness and hydrophobicity increase, after which there is a decrease in these parameters. This behavior suggests that up to a critical amount of TEOS, the results are influenced by the morphology and topographical features, after which a major role seems to be played by surface chemistry—increasing the oxygenation surfaces. The morphological and chemical details and also the hydrophobicity/hydrophilicity characteristics are discussed in the attempt to design biological surfaces with optimal wettability properties and possibility of application in tissue engineering.

## 1. Introduction

Recently, progress has been made in tissue engineering and regenerative medicine concerning the design and manufacture of scaffolds for cell growth and development used in tissue repair. Tissue engineering uses biological scaffolds in order to restore, maintain and replace diseased tissues whose functions have been affected. The polymers have gained attention in the obtaining of these biomaterials due to the synthesis flexibility, variety of functional groups, specific physical properties as strength and hardness, hydrophilic/hydrophobic features and especially biocompatibility. Among these, natural polymers as proteins (silk, collagen, fibrinogen, elastin), polysaccharides (cellulose, chitosan, dextran) or polynucleotides (DNA, RNA) were the first used in the clinical field due to their bioactive properties which consist of good interaction with cells whose performance improves. As polymer-containing glucose groups, cellulose seems to be one of the most suitable due to the versatility of its physical and chemical characteristics [1]. However, given that the workability of this polymer is limited by low solubility in different solvents or that some cellulosic materials do not have specific properties that meet the requirements of daily life, the development of new compounds/systems based on cellulose with improved characteristics is pursued. Thus, through chemical (introduction of functional groups) or physical changes (mixing with various compounds) it will be possible to obtain new high-performant cellulosic materials of different forms (hydrogels, films, fibers). These materials will find applicability in various fields including the medical one as drug delivery systems, prosthetic devices or scaffolds for cell culture [2,3]. There is a continuous interest and also a challenge for researchers in the use of cellulose materials and especially of the cellulose materials surfaces due to the physico-chemical processes that take place at the cellulose/cellulose interface but also cellulose/different media.

Mixing of cellulose or cellulose derivatives with various inorganic materials such as silica, titan, or alumina leads to different reorganizations of macromolecular chains that have an impact on the final properties of cellulosic materials directing them to certain applications [4,5,6]. In order to obtain cellulosic materials with specific properties required by applications aimed at growth and cell development, the present study chose cellulose acetate (CA) and tetraethyl orthosilicate (TEOS)—a precursor for obtaining silica nanoparticles. Cellulose acetate is the most common organic cellulose ester, a cost-effective biodegradable polymer, often used in the form of fibrous materials or coatings. Previous studies have shown that cellulose acetate has the ability to form membranes with pores of different shapes and sizes [7,8]. This property is of real interest in tissue engineering because it will allow cell attachment and also will favor the diffusion of the nutrients and metabolites throughout the volume [9]. Moreover, as the literature mentions, CA-based nanocomposites were used in medical applications as membrane for dialysis—component part of the artificial kidney [10], for applications that require superior clarity—optical sensors [11], in controlled release of actives and enzymes immobilization, in controlled release of drugs, and in the field of bone repairing and tissue engineering [12,13,14]. One of the most important conditions for using cellulosic materials in tissue engineering is that the mechanical properties match with those of the host tissue. In the case of bone tissue engineering, the restoration of biomechanical functions is crucial and, therefore, the polymeric bioscaffolds must have similar properties with natural bones and a degradation behavior that corresponds to the rate of new bone formation [15]. For this reason, the challenge is to improve these properties, one of the ways being the mixture of the cellulose acetate with inorganic compounds [16,17,18]. As was mentioned, TEOS was chosen because of its ability to form silica nanoparticles that will enhance the mechanical properties and stability in terms of the chemical agent action and thermal stability of cellulosic materials but also will improve the surface roughness and adhesion properties. In addition, from literature data, it seems that the silica nanoparticles present also biocompatibility properties that depend on their synthesis method and make the silica-based nanomaterials to be suitable for dental and skeletal applications [19,20,21]. Previous studies have shown that the sol-gel method offers the advantage of obtaining silica nanoparticles of certain forms that can be uniformly dispersed in different environments, important factors in biocompatibility and reproducibility [22]. The silica nanoparticle appearance will lead to changes of properties, in the mass of cellulosic films (mechanical resistance, thermal stability), but also will have an impact on their surface features (roughness, wettability, adhesion) and surface chemical structure [23,24,25]. Although the changes in the functional properties of cellulose due to the use of silica-containing compounds have been presented in the literature, this topic continues to be a challenge for researcher. For this reason, unlike the studies previously performed [16,17,18], this paper represents an in-depth experimental research based on structural and topological techniques achieved in order to evaluate the applicative potential of cellulose/silica systems as membranes for bone cell growth and development. In this context, cellulose acetate/silica composite films were investigated concerning their surface characteristics and biocompatibility. These properties need to be known for design of porous surfaces with topographies that favor cell growth and development, and to avoid the deficient integration of tissue in the living organism. Thus, the data of the present studies have the role to predict which of the analyzed cellulose acetate/silica films will have the most suitable surface properties (morphology, topography), wettability and biocompatibility to be used as bioscaffolds in tissues engineering.

## 2. Materials and Methods

### 2.1. Materials

Cellulose acetate (CA) with a molecular weight of Mn = 50,000 g/mol, and an acetyl content between 39.2 and 40.2 wt.% which corresponds to a substitution degree of DS = 2.44 was purchased from Sigma–Aldrich, St. Louis, MO, USA, and used as such, without further purification.

Tetraethyl orthosilicate (TEOS) reagent grade, 98%, hydrochloric acid (HCl) aqueous solutions 37%, and tetrahydrofuran (THF) (molar weight of 72.110 g/mol), were also purchased from Sigma–Aldrich, USA, and used without previous purification.

The films subjected to analysis were cast from CA/TEOS solution (in different mixing ratios) of 10 g/dL concentration, obtained through sol-gel technique that allows the formation of silica nanoparticles—protocol presented in detail in a previous study [22].

### 2.2. Methods for Sample Characterization

Atomic force microscopy (AFM) images were recorded in air, in a tapping mode, on a scan area of $10\times 10{\;\mu m}^{2}$, using an NTEGRA Spectra (NT-MDT, Russia) instrument with 3.1–37.6 $N\cdot m^{-1}$ force constant cantilever of a silicon nitride cantilevers (NSC10, NT-MDT, Moscow, Russia). The differences in film surface morphology were expressed in terms of the average roughness, Ra, and root mean square of roughness, Rq. Statistical data of the particle size distributions were obtained in Nova software by measuring 60 nanoparticles for each film.

X-ray Photoelectron Spectroscopy measurements, using a Physical Electronics PHI 5000 VersaProbe instrument, UK, were carried out to determine the surface elemental composition of the samples. The XPS system was equipped with mono-chromated Al Kα X-ray source (1486.7 eV). The calibration of the binding energy (BE) scale was performed by considering the BE of the C 1s peak (284.6 eV) from the carbon contamination layer. Peak deconvolution has been done using PHI-MultiPak software. The calculation software measures values of atomic concentration of ±0.1%.

To assess the wettability of the samples, static contact angles were measured by sessile drop technique at room temperature, using CAM 101 Optical Contact Angle instruments (KSV Instruments Ltd., Helsinki, Finland). Images were recorded with a special optical system equipped with a CCD camera connected to a computer. The test liquids used for determinations were MilliQ water, ethylene glycol and diiodomethane. A drop of liquid (~1 μL) was placed, with a Hamilton syringe, on a specially prepared plate of substratum and the image was immediately sent via the CCD camera to the computer for analysis. All the measurements were done in triplicate and the results were recorded as mean ± standard deviation.

The stress/strain curves were recorded on a Shimadzu AGS-J deformation apparatus at ambient temperature and at a rate of deformation of 10 $\mathrm{mm}\cdot \min^{-1}$ with a load cell capable of measuring forces up to 1 kN and a sample film of 20 mm × 6 mm × 0.13 mm. For each data point, five measurements were made, and the average value was taken.

Moisture sorption capacity of the samples has been determined in dynamic regime, using a fully automated gravimetric device IGAsorp made by Hiden Analytical (Warrington, UK). The essential part of this equipment is an ultrasensitive microbalance which measures the weight change as the humidity is modified in the sample chamber at a constant temperature. The measurements are controlled by a user-friendly software package. After the samples were placed in a special container, they were dried at 25 °C in flowing nitrogen (250 $\mathrm{mL}\cdot \min^{-1}$) until their weights were in equilibrium at a relative humidity, RH, less than 1%. Then, the RH was gradually increased from 0 to 90%, in 10% humidity steps, every having a pre-established equilibrium time between 10 and 20 min and the sorption equilibrium was obtained for each step. The RH was decreased and desorption curves were registered. For each data point, five measurements were made, and the average value was taken.

## 3. Results

### 3.1. Morphological and Topographical Aspects of Cellulose Acetate/Silica Films

**Figure 1:**
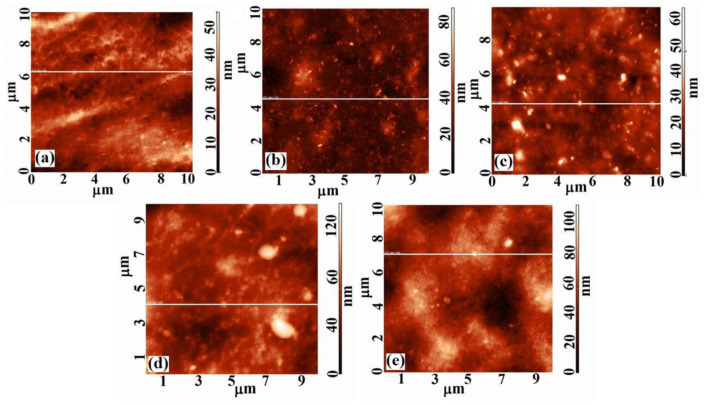
2D AFM images obtained over $10\times 10{\;\mu m}^{2}$ scan area for (**a**) CA, (**b**) CA+0.5 wt.% TEOS, (**c**) CA+1.0 wt.% TEOS, (**d**) CA+1.5 wt.% TEOS and (**e**) CA+2.0 wt.% TEOS films.

**Figure 2:**
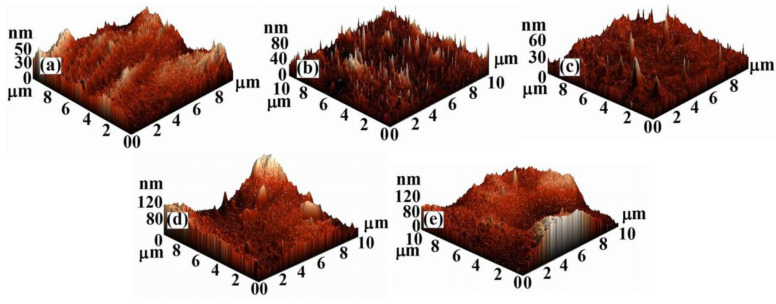
3D AFM images obtained over $10\times 10{\;\mu m}^{2}$ scan area for: (**a**) CA, (**b**) CA+0.5 wt.% TEOS, (**c**) CA+1.0 wt.% TEOS, (**d**) CA+1.5 wt.% TEOS and (**e**) CA+2.0 wt.% TEOS films.

**Figure 3:**
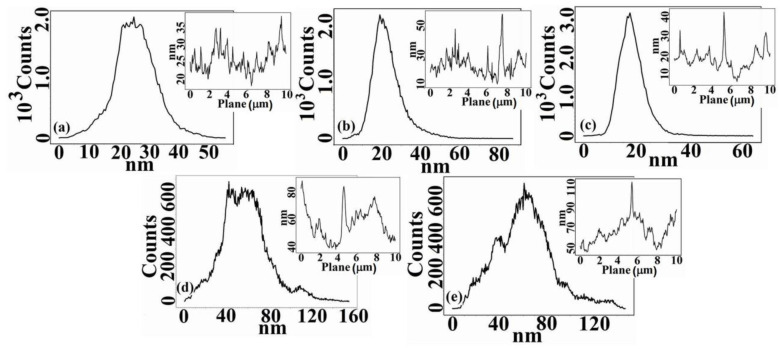
Histograms and surfaces profiles (small plots) taken along a line from 2D AFM images of: (**a**) CA, (**b**) CA+0.5 wt.% TEOS, (**c**) CA+1.0 wt.% TEOS, (**d**) CA+1.5 wt.% TEOS and (**e**) CA+2.0 wt.% TEOS films.

The literature data show that the properties in solution, dictated by the configuration and conformation of the polymeric chains, influence their final rearrangement/reorganization in the solid state and, implicitly, the morphology and topography of the corresponding film surfaces [26,27]. Thus, the cellulose acetate/silica composite films were investigated through AFM analysis in order to obtain information concerning the interactions that occur in the studied systems, and to understand the hydrolysis and condensation processes specific to the protocol for solutions obtaining [22]. These processes lead to apparition of the silica nanoparticles that will also have an impact on the films surfaces characteristics. The 2D, 3D AFM images and the corresponding profiles and histograms (Figure 1, Figure 2 and Figure 3) obtained for $10\times 10{\;\mu m}^{2}$ scan area show that the addition of TEOS in the CA casting solution has a major impact on the morphological characteristics and topography of the corresponding dried films, through the interaction that it generates in the systems.

The surfaces are characterized by heights and valleys, but also by aggregates/agglomerations of different shapes and sizes. On the surface of CA films, areas with nodules of high widths are highlighted. The widths of these nodules decrease with addition of TEOS (Figure 2) until a content of 1.0 wt.%, after which increase again. Moreover, the surface of the CA film is smoother than that of the samples containing TEOS. It is observed that the film’s roughness increases very slightly up to a content of 1.0 wt.% TEOS after which the increase becomes sudden (Table 1). This can be explained by the fact that –OH groups of the CA are replaced by the voluminous –CH_3_ groups of TEOS during the process of casting solution obtaining [22]. New bonds that appear in the systems influence the rearrangement of the macromolecular chains both in the mass and on films surfaces leading to an increase in roughness. Consequently, the surfaces of the samples containing 0.5 and 1.0 wt.% TEOS are characterized by numerous nodules of small widths, but whose heights increase, the roughness varying slightly compared with that of CA sample. Starting with 1.5 wt.% TEOS the widths of nodules begin to increase, the roughness of the surfaces reaching the highest values (Figure 3, Table 1).

**Figure 4:**
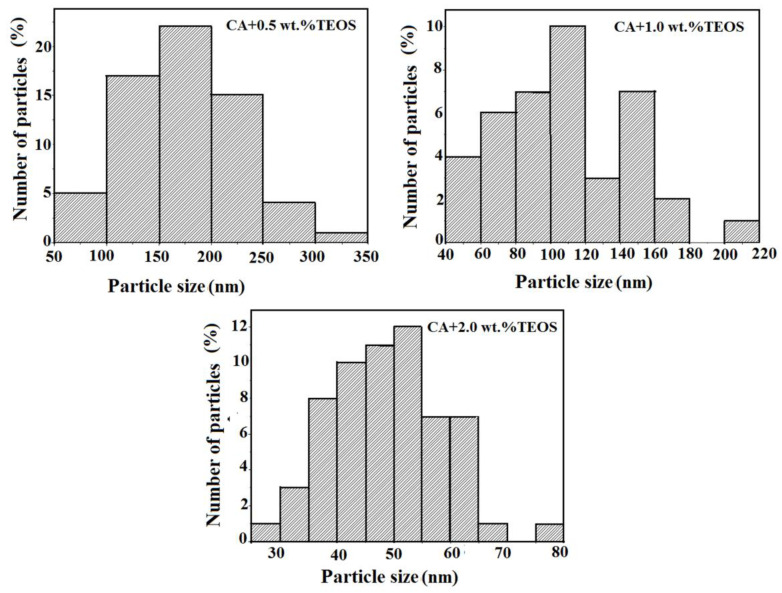
Particle size distributions obtained based on 2D AFM images for cellulose acetate/silica films.

The variation of the surface characteristics of the studied films can be attributed also to the silica nanoparticles. These were obtained as a result of the hydrolysis and condensation processes that take place during the CA/TEOS solutions obtaining and, implicitly, of the main interactions that occur in the systems. It is known that the silica nanoparticles containing silanol groups, have a hydrophilic character and present numerous binding sites [28]. These will favor the chemical bonding capacity and appearance of irregular agglomerates which is reflected in AFM images. The statements are in agreement with rheological data reported previously [22], where was observed that, as TEOS content from the initial CA solutions increase, an increase in viscosity occurs, as a result of the apparition of aggregates, generated in turn, by hydrogen or covalent bonds. For low contents of TEOS is especially favored the apparition of hydrogen bonds between the –OH groups of the CA chains, and less favored those between the –OH groups of the CA chains and of silanol groups (Si–OH) belonging to TEOS. Comparatively, for high content of TEOS the interactions that predominate and are more intense are those established between the –OH groups of the CA chains and those of Si-OH groups, and that for 2.0 wt.% TEOS determine the appearance of the gel network. Therefore, in evaluating the surface’s properties, it is necessary to take into account both the interactions that occur during the solutions obtaining process, but also the existence of silica nanoparticles. As can be seen from the AFM images (Figure 1 and Figure 2), the silica nanoparticles do not have a well-defined shape, their number increases as the amount of TEOS from the film’s casting solution increases, and their dimensions decrease from 170 nm (0.5 wt.% TEOS) to 50 nm (2.0 wt.% TESO) (Table 1), being comparable to those found in the literature [29]. These observations can be deduced from the distribution of particles number function on their dimensions; for a maximum content of TEOS a greater distribution of particles by size exist (Figure 4). Moreover, in Figure 1b,c is observed a favorable distribution of silica nanoparticles, an agglomeration of them being visible for high TEOS content (Figure 1d,e).

The irregular distribution of the silica nanoparticles at films surfaces can be explained by behavior in a concentrated solution. The tendency to gelling of the CA/TEOS solution that begins to be felt from 1.5 wt.% TEOS and, implicitly, the gel network, that appears for maximum content of TEOS [22], prevent the diffusion of silica nanoparticles leading to non-uniformity in their distribution and, consequently, to an increase of the corresponding film’s surface roughness. Thus, it can be assumed that the performance of the films/membranes in which the silica nanoparticles are uniformly dispersed is higher than that of films/membranes with patchy distribution. Therefore, the film’s surface morphology dictated by the dispersion mode of the silica nanoparticles has a great impact on their applications as composite membranes in different fields of activity [30,31,32].

### 3.2. Qualitative and Quantitative Surface Investigations of Cellulose Acetate/Silica Films

Generally, many of the polymeric material properties are established by the composition of their surfaces, this composition differing from the bulk. Moreover, for composite materials in which metallic phases are incorporated, the chemistry of the surface and interface with the important role in assigning the material properties is necessary to be known.

Thus, XPS analyzes were performed in order to know, in detail, the chemistry of the cellulose acetate/silica composite films surfaces, an important parameter in obtaining biomaterials with cell development function. In this sense, the films were subjected to the action of a soft electromagnetic radiation, under high vacuum, the atomic layers from the surfaces being the most sensitive. As a result of this analysis, the percentages of C, O, and Si from films surfaces were determined, the results being listed in Table 2.

According to the obtained data, it is observed that the addition of TEOS in systems causes an increase in Si content. At the same time, the percentage of oxygen increases as a result of the enrichment with oxygen belonging to TEOS, but also of the air exposure of sample, while the level of carbon due to carbon contaminants decreases. These results indicate that the arrangements in the casting solutions tend to form a SiO_2_-like structure [33,34].

**Figure 5:**
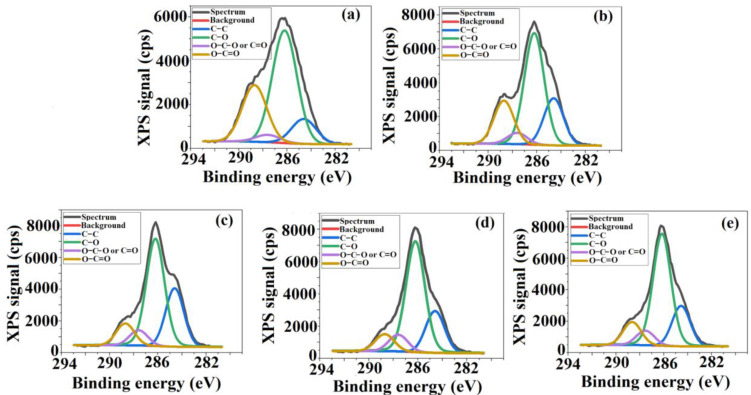
The deconvolution of C 1s spectra for: (**a**) CA, (**b**) CA+0.5 wt.% TEOS, (**c**) CA+1.0 wt.% TEOS, (**d**) CA+1.5 wt.% TEOS, (**e**) CA+2 wt.% TEOS films.

**Figure 6:**
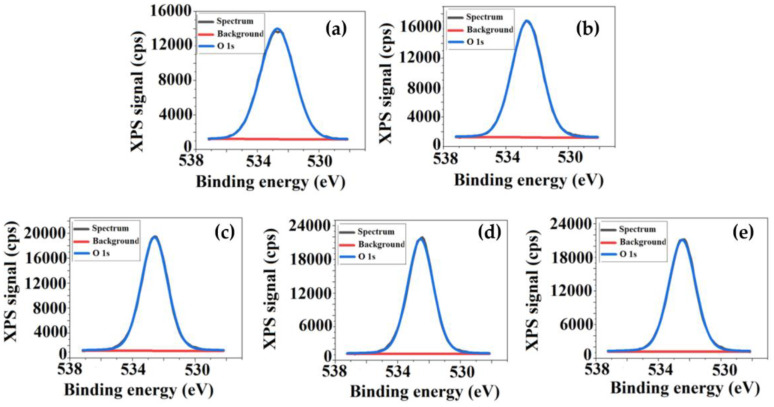
The deconvolution of O 1s spectra for: (**a**) CA, (**b**) CA+0.5 wt.% TEOS, (**c**) CA+1.0 wt.% TEOS, (**d**) CA+1.5 wt.% TEOS, (**e**) CA+2 wt.% TEOS films.

**Figure 7:**
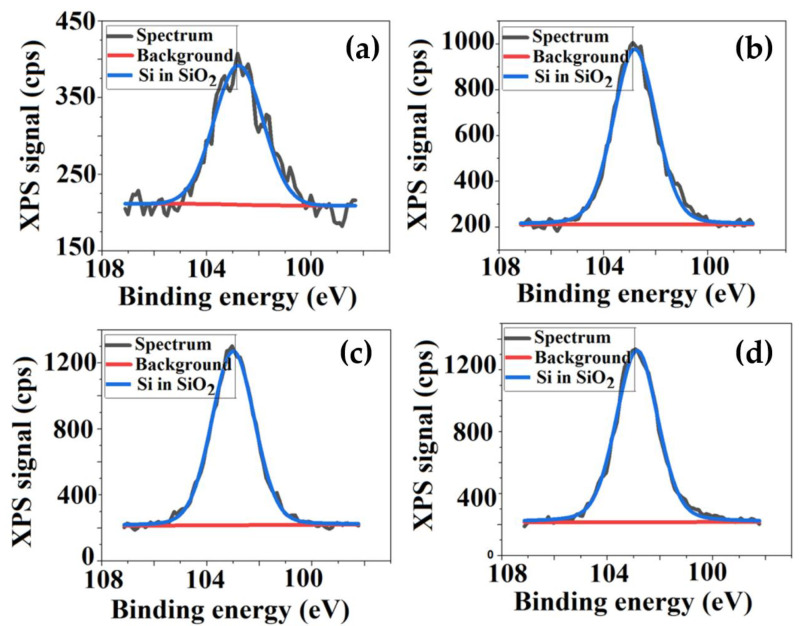
The deconvolution of Si from SiO_2_ spectra for: (**a**) CA+0.5 wt.% TEOS, (**b**) CA+1.0 wt.% TEOS, (**c**) CA+1.5 wt.% TEOS, (**d**) CA+2 wt.% TEOS films.

Variation of TEOS content determines some changes both in atomic percentage of the elements, but also in the number and intensities of the corresponding peaks observed in the XPS spectra. Therefore, besides the peaks specific to CA sample, assigned to C 1s and O 1s located at 286.5 eV and 533 eV respectively, new peak around 103 eV was attributed to Si 2p. This indicates that TEOS, which was incorporated into the cellulose matrix by sol-gel technique—a method that consists in the hydrolysis and condensation of an alkoxide in the presence of an acid—leads to the in situ formation of silica nanoparticles (SiO_2_). Thus, it can be explained the bonding of Si to the surfaces of polymeric films [22,35]. In order to have a clearer picture of the chemical states, the deconvolutions of the spectra of each element were performed, Figure 5, Figure 6 and Figure 7 represent the fitted C 1s, O 1s and Si spectra for the analyzed samples. The C 1s spectrum (Figure 5) can be deconvoluted to four peaks centered at 284.9 eV, 286 eV, 287.5 eV and 289 eV, and can be assigned to C–C, C–O, O–C–O or C=O and O–C=O respectively, the results being very close to those found in the literature [36,37].

The O 1s and Si 2p spectra are fitted to a single contribution; that of O 1s corresponds to a binding energy of 533 eV, while of Si 2p to 103 eV, the latter highlighting the emergence of new types of interactions such Si–O–C and Si–O–Si as TEOS was added in the system [35,38,39].

The apparition of new types of interactions, as well as the increase of the O and Si percentage from 38.7% to 43.1% and 0.6% to 4%, respectively, confirms the existence of silica nanoparticles on the analyzed film surfaces. In addition to those mentioned it was observed that the intensity of all peaks from XPS spectra increases. This increase is a consequence of the increase in the concentration of the atomic species, as TEOS content from the systems becomes higher, and also to the sensitivity of each of them as a result of exposure to electromagnetic radiation. The latter is related to their oxidation state: a high binding energy corresponding to a high oxidation state.

Consequently, the XPS results, through the spectral peaks, confirm the successful incorporation of TEOS into the cellulosic matrix, providing information about the membrane’s surface chemistry and, implicitly, silica nanoparticle apparition. The presence of silica nanoparticles on the polymeric surfaces is of real help for the desired biomedical application, namely the growth and proliferation of bone cells. As the literature mentions, these nanoparticles can support bone cell differentiation by improving bone mass [40,41,42].

### 3.3. Wetting Properties of Cellulose Acetate/Silica Films

**Figure 8:**
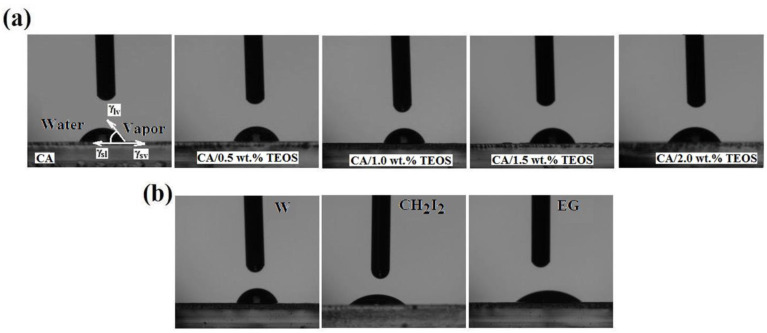
(**a**) Geometric configuration of water liquid droplets and the surface forces acting at the cellulose acetate/silica-water interface and (**b**) geometric configuration of used liquid droplets for CA+1 wt.% TEOS film.

Knowledge of the polymeric surface wettability properties play an important role in the design of materials that can be used in the medical field, especially for targeted application. Tests such as biocompatibility, adhesion, growth, and cell development are performed in aqueous environments and, for this reason, control of the hydrophilic/hydrophobic balance of the materials designed for this purpose is required. However, the cell type has also an important role through the nature of the composition, cell wall structure, but also through the forces that take place between them and polymeric surfaces. Taking into account these aspects, it is necessary to find the optimal wettability parameters that ensure cell growth and development. As is known, the wetting properties of the polymeric surfaces are given by the interactions that are established at the solid-liquid interface, and that appears as a result of the physical contact between the two phases. At the fundamental level, the nature of these interactions is of atomic origin and represents the energy balance between intermolecular, electrostatic, and gravitational energy. As a result of these energies, a drop of liquid that comes in contact with the solid surface will acquire a geometric configuration that mimics the total energy of the solid-liquid system [43]. In the present work, the liquids used for the contact angle measurements were water (W), diiodmethane (CH_2_I_2_), and ethylene glycol (EG). Depending on the surface tensions of each liquid and the energies developed through specific forces, the shape of the drops change (Figure 8).

As can be seen (Figure 8a), for samples containing up to 1.0 wt.% TEOS, the water drop changes its shape from flat to round. This means that the silica nanoparticles and rearrangement of macromolecular chains at films surfaces lead to an increase in roughness, contact angle values, and implicitly, of the hydrophobicity. This aspect is sustained by literature data, according to which, the processes that take place in the mass of the solution—in the initial stage of obtaining (acetylation, sol-gel processes, or silica grafting) —lead to an increase in hydrophobicity [44]. For films whose TEOS content exceeds 1.0 wt.%, the drop flattens and is more easily absorbed highlighting a hydrophilic behavior of the samples. Therefore, from a critical content of TEOS the film surfaces are enriched in oxygen, and at the same time absorb oxygen as result of air exposure, which will lead to an increase in hydrophilicity.

From Table 3 and Figure 8b can be observed the way in which varies the contact angles values and shapes of the used liquid drops for samples containing 1 wt.% TEOS. As is observed, the geometric configuration of the liquid drops modifies depending on the specific parameters of the liquids (superficial tensions) and forces that they determine. As literature mention for a contact angle ranging between 10°<θ<90° the polymeric surfaces is hydrophilic, while for 90°<θ<150° are hydrophobic, superhydrophilicity and superhydrophobicity appearing when θ<10° and θ>150°, respectively [43,45]. In addition, according to some studies in the tissue engineering field, it is assumed that the polymeric surfaces for which the contact angles values vary between 50–70° are the most suitable for cell adhesion [46].

According to these literature data, the analyzed surfaces seem to be suitable for the desired application. However, taking into account the differences that appear at the morphological and structural level, optimal blend compositions are necessary to find. Therefore, in order to establish the hydrophilic/hydrophobic character and to anticipate, through theoretical approximations, the biocompatibility of the CA/TEOS composite films, the knowledge of the corresponding surface tension parameters is required. In this sense, the acid/base method (LW/AB) (Equations (1)–(4)) [47,48,49,50,51,52,53], which involves the surface tension parameters of test liquids (Table 4) [47,48,49,50,51,52,53] and values of the contact angles (Table 3) measured between the solid surface and used liquid drops, was applied.

(1) $$1+\cos\theta =\frac{2}{\gamma_{\mathrm{lv}}}\cdot \left(\sqrt{\gamma_{\mathrm{sv}}^{d}\cdot \gamma_{\mathrm{lv}}^{d}}+\sqrt{\gamma_{\mathrm{sv}}^{+}\cdot \gamma_{\mathrm{lv}}^{-}}+\sqrt{\gamma_{\mathrm{sv}}^{-}\cdot \gamma_{\mathrm{lv}}^{+}}\right)$$

(2) $$\gamma_{\mathrm{sv}}^{p}=2\cdot \sqrt{\gamma_{\mathrm{sv}}^{+}\cdot \gamma_{\mathrm{sv}}^{-}}$$

(3) $$\gamma_{\mathrm{sv}}=\gamma_{\mathrm{sv}}^{d}+\gamma_{\mathrm{sv}}^{p}$$

(4) $$\gamma_{\mathrm{sl}}={\left(\sqrt{\gamma_{\mathrm{sv}}^{d}}-\sqrt{\gamma_{\mathrm{lv}}^{d}}\right)}^{2}+2\cdot \left(\sqrt{\gamma_{\mathrm{sv}}^{+}\gamma_{\mathrm{sv}}^{-}}+\sqrt{\gamma_{\mathrm{lv}}^{+}\gamma_{\mathrm{lv}}^{-}}-\sqrt{\gamma_{\mathrm{sv}}^{+}\gamma_{\mathrm{lv}}^{-}}-\sqrt{\gamma_{\mathrm{sv}}^{-}\gamma_{\mathrm{lv}}^{+}}\right)$$

where:

- θ represent the contact angle between the test liquids and polymeric surface;
- “lv” and “sv” are subscripts that denote the liquid-vapor and surface-vapor interfacial tension, respectively;
- “p” and “d” are superscripts that denote the polar and disperse components, respectively, of total surface tension;
- $\gamma_{\mathrm{sv}}$ is the total surface tension.

The data obtained for surface tension parameters (Table 5) show that, for all samples, the disperse components, $\gamma_{\mathrm{sv}}^{d}$, are always higher than the polar one $\gamma_{\mathrm{sv}}^{p}$, while the electron acceptor parameter, $\gamma_{\mathrm{sv}}^{+}$, is smaller than the electron donor parameter, $\gamma_{\mathrm{sv}}^{-}$.

**Table 4:** Surface tension parameters of the liquids used [47,48,49,50,51,52,53].

Liquid	$\gamma_{\mathrm{lv}}$	$\gamma_{\mathrm{lv}}^{d}$	$\gamma_{\mathrm{lv}}^{p}$	$\gamma_{\mathrm{lv}}^{+}$	$\gamma_{\mathrm{lv}}^{-}$
Water (W)	72.8	21.8	51.0	25.5	25.5
Diiodmethane (CH_2_I_2_)	50.8	50.8	0.0	0.72	0
Ethylene glycol (EG)	48.0	29.0	19.0	1.92	47.0
Red blood cells	36.56	35.20	1.36	0.01	46.2
Platelets	118.24	99.14	19.10	12.26	7.44
Albumin	62.50	26.80	35.70	6.30	50.60
Fibrinogen	41.50	37.60	3.89	0.10	38.00
Immunoglobulin (IgG)	51.30	34.00	17.30	1.50	49.60

**Figure 9:**
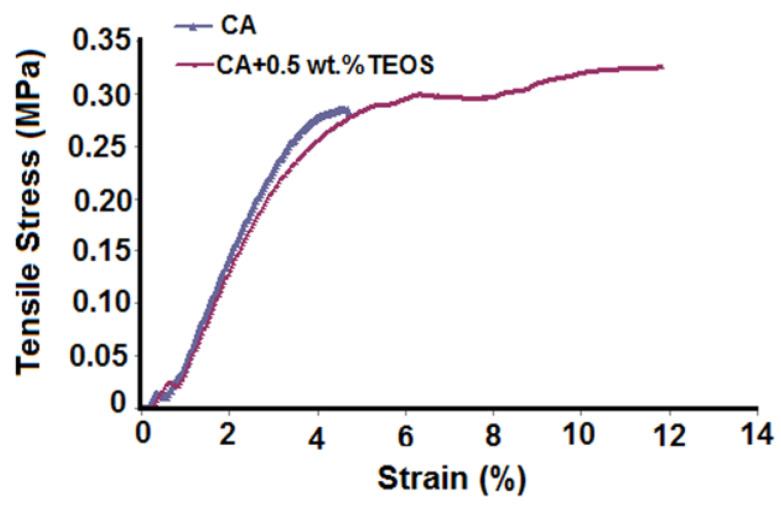
Tensile strength of CA and CA+0.5 wt.% TEOS samples versus applied strain.

Analyzing the results depending on TEOS content it is observed that the polar component which dictates the hydrophilic character of the studied sample varies. Thus, compared to CA film, for which the polar component is high, for the samples whose TEOS content increases, a slight decrease in this parameter is observed. This happens up to 1.0 wt.% TEOS after which, an increase of the polar component occurs. As was mentioned, the introduction of TEOS in systems is responsible for generating new interactions (hydrogen or covalent bonds), and also of silica nanoparticles, these representing factors greatly influencing the wetting properties. For low TEOS contents, the increase of roughness and relatively uniform distribution of the silica nanoparticles at films surfaces lead to a decrease of the hydrophilicity. In addition, according to data from previous studies [22] for high TEOS contents, the interactions that appear in the systems especially the covalent ones, will generate a high number of silica nanoparticles which, in turn, will lead to non-uniform and brittle films. This statement is sustained by data obtained from tensile strength measurements (Figure 9), that can be performed only up to a content of 1 wt.% TEOS. The data showed that the samples behave as rigid brittle solids. CA sample fractures right after the yield strength point, at 0.25 MPa stress and 5% strain. Sample containing 0.5 wt.% TEOS has yielded strength similar to the CA sample, followed by an extended region of strain hardening. The capability of the sample with a small content of TEOS to absorb more energy before breaking is due to the reinforcing network of silica formed by hydrolysis of TEOS introduced in the formulation used for the preparation of the sample. This is reflected in the difference for the ultimate toughness of 0.088 $\mathrm{kJ}\cdot m^{-3}$ for CA film and 0.94 $\mathrm{kJ}\cdot m^{-3}$ for the sample with 0.5 wt.%TEOS.

Starting with a content of 1.5 wt.%, although the roughness is high and the hydrophobicity is expected to increase, due to changes in surface chemistry, oxygen enrichment, but also to the non-uniform arrangement of the silica nanoparticles, there is an increase in samples hydrophilicity. Moreover, the silica nanoparticles with their binding characteristics generated by the hydroxyl groups, belonging to silanols, determine an increase of the polar component. So, up to a critical content of 1.0 wt.% TEOS, the roughness dictates the hydrophilic/hydrophobic character of the samples, after which the surface chemistry is the one that has an important impact on this parameter.

**Figure 10:**
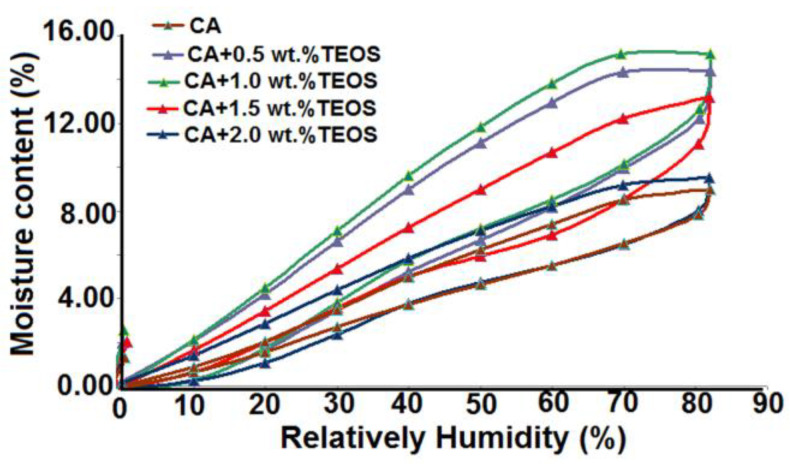
Sorption/desorption isotherms for the studied samples.

In addition to the above-mentioned results, based on the water vapor sorption data (Figure 10, Table 6), it was shown that the film’s permeability, reflected in the values of water vapor sorption capacities, depends on both material structure and hydrophilic/hydrophobic features. Moreover, the results on their surfaces highlight the existence of nano-sized pores, whose number varies from sample to sample, depending on the structural peculiarity of polymer and TEOS composition from the blends.

Consequently, according to those above mentioned, but also to the previous research in concentrated solution [22] it can be stated that solutions containing small amounts of TEOS will facilitate not only the obtaining of membranes/films and analyzing of their surfaces from morphological and topographical point of view, but also the acquiring of viable data related to wetting. This property can be evaluated also by surface free energy. Conform literature data, a decrease of the hydrophilicity can be determined, on the one hand, by the surface-free energy decrease—as a result of the surface chemistry changes and, on the other hand, by the increase of roughness [53,54]. In the present study, the addition of TEOS leads to changes in surface chemistry through the interactions that it generates in the systems. These interactions will influence the orientation of the macromolecular chains in the casting solution, modifying the chemical structure at the corresponding films’ surfaces. For low contents of TEOS, the interactions between the –OH groups of CA predominate to the detriment of those between the –OH groups of CA and of the silanol groups (Si–OH) belonging to TEOS leading to modifications in surface chemistry, but also to a slight increase in roughness. For the high content of TEOS, the latter mentioned interactions become more significant causing considerable variations both in surface chemistry (see Figure 5, Figure 6 and Figure 7) and topography.

In the context of the chemical and morphological changes, the surface free energy, $\Delta G_{w}$, also modifies dictating the hydrophilic/hydrophobic character of the samples. This parameter was calculated based on Equation (5) [47] using $\gamma_{\mathrm{lv}}$ and $\theta_{\mathrm{water}}$ from Table 3 and Table 4.

(5) $$\Delta G_{w}=-\gamma_{\mathrm{lv}}\cdot (1+\cos\theta_{\mathrm{water}})$$

**Figure 11:**
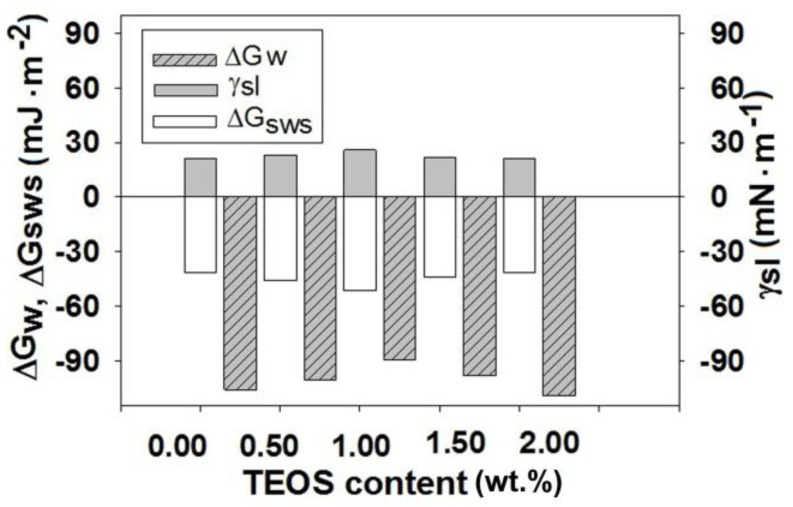
The surface free energy, solid–liquid interfacial tensions, and interfacial free energy versus TEOS content, for cellulose acetate/silica films evaluated taking into account water contact angle.

The variation of $\Delta G_{w}$ as function on TEOS content is represented in Figure 11.

According to literature, the samples that present $\Delta G_{w}<-113$ $\mathrm{mJ}\cdot m^{-2}$ are considered to be hydrophilic, while those with $\Delta G_{w}<-113$ $\mathrm{mJ}\cdot m^{-2}$ hydrophobic [54]. Thus, in our case, the obtained results denote a slightly hydrophobic character of the films. However, evaluating function on TEOS content, this parameter changes following the same variation as the polar component. For low contents of TEOS, the values of the surface free energy increase compared with that of CA sample while starting with 1.5 wt.% TEOS, a decrease is observed. This means that samples with high amount of TEOS have a higher wetting capacity. In addition to those mentioned, for cellulose acetate/silica composite films containing 1.5 and 2.0 wt.% TEOS the large number of silica nanoparticles that appear on surfaces may suggest the existence of interfacial tensions that are established between them and polymeric matrix and that have also impact on wetting parameter.

The influence of TEOS addition in the CA casting solutions on wettability properties of corresponding films can also be explained by the solid-liquid interfacial tensions, $\gamma_{\mathrm{sl}}^{}$, interfacial free energy, $\Delta G_{\mathrm{sws}}^{}$, between two particles of cellulose acetate in water (Equations (6) and (7) [47]) and by work of spreading of water, $W_{s,w}$, implicitly (Equation (8) [48]).

(6) $$\Delta G_{\mathrm{sws}}^{}=-2\cdot \gamma_{\mathrm{sl}}$$

(7) $$\gamma_{\mathrm{sl}}={\left(\sqrt{\gamma_{\mathrm{lv}}^{p}}-\sqrt{\gamma_{\mathrm{sv}}^{p}}\right)}^{2}+{\left(\sqrt{\gamma_{\mathrm{lv}}^{d}}-\sqrt{\gamma_{\mathrm{sv}}^{d}}\right)}^{2}$$

(8) $$W_{s,w}=W_{a}-W_{c}=2\cdot [{\left(\gamma_{\mathrm{sv}}^{\mathrm{LW}}\cdot \gamma_{\mathrm{lv}}^{d}\right)}^{1/2}+{\left(\gamma_{\mathrm{sv}}^{+}\cdot \gamma_{\mathrm{lv}}^{-}\right)}^{1/2}+{\left(\gamma_{\mathrm{sv}}^{-}\cdot \gamma_{\mathrm{lv}}^{+}\right)}^{1/2}]-2\cdot \gamma_{\mathrm{lv}}$$

where values for $\gamma_{\mathrm{lv}}^{p}$, $\gamma_{\mathrm{lv}}^{d}$, $\gamma_{\mathrm{sv}}^{p}$ and $\gamma_{\mathrm{sv}}^{d}$ are tabulated in Table 4 and Table 5, respectively.

$W_{s,w}$ represents the difference between the work of water adhesion, $W_{a}$, and work of water cohesion, $W_{c}$.

The variation of the interfacial free energy, $\Delta G_{\mathrm{sws}}^{}$, function of TEOS content is also represented in Figure 11. As is observed from this dependence the values obtained for $\Delta G_{\mathrm{sws}}^{}$ are negative confirming that the sample is less hydrophilic, meaning that between the two polymer surfaces, s, immersed in water, w, an attraction occurs.

**Figure 12:**
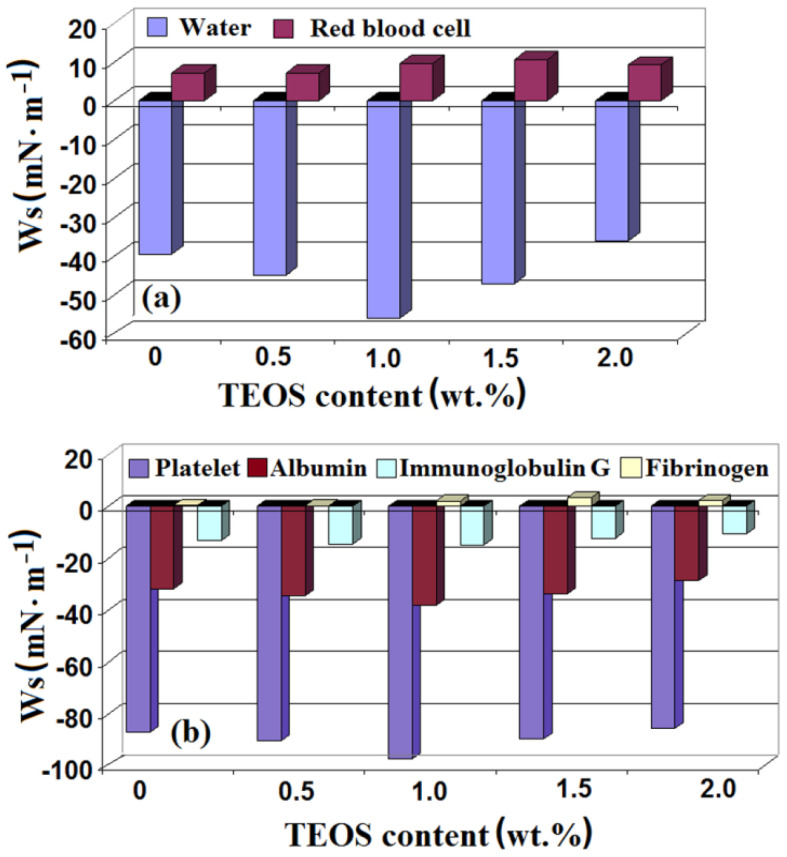
Work of spreading of (**a**) water, red blood cells, (**b**) platelets, and plasma proteins versus TEOS content for cellulose acetate/silica films.

Also, the work of spreading of water, $W_{s,w}$, over the cellulose acetate/silica surfaces show samples slightly hydrophobic (Figure 12).

The negative values obtained for $W_{s,w}$ suggest that the work of water adhesion is lower compared with works of water cohesion. However, from sample to sample, the values of the interfacial free energy, as well as those for work of spreading of water present the same variation as polar component and surface free energy. The small values of $W_{s,w}$ obtained for samples with low content of TEOS denote an increase in hydrophobicity, while slightly increased values of $W_{s,w}$ for samples with high content of TEOS is associated with an increase in hydrophilicity.

Thus, based on the results obtained from morphological analysis and those above presented the most suitable mixtures for the targeted biomedical application seems to be those containing up to 1.5 wt.% content of TEOS.

### 3.4. Blood Components and Plasma Proteins Interaction with Cellulose/Silica Surfaces

In general, the applicability of polymeric materials in the biomedical field in the form of any device that comes into contact with the living organism is limited by their biocompatibility properties. The first fluid that interacts with the biomaterial is blood, and, for this reason, the polymeric surface-blood interaction is important to be evaluated. Once inside the body, on the surface of the biomaterial there are processes of plasma protein adsorption, platelet adhesion and activation, coagulation and thrombosis, processes that will dictate the biomaterial lifetime. Thus, in the present work, before in vitro studies concerning the usage of cellulose acetate/silica membranes as scaffolds in tissues engineering, a theoretical estimation of the biocompatibility has been made. In this sense, it was necessary to determine the spreading work of the blood components and different plasma proteins over the polymeric surfaces by using tension parameters listed in Table 4 and Table 5 and Equation (8). Data obtained were represented in a graph showing the variation of the work of spreading as a function on TEOS content (Figure 12).

As can be seen, the work of spreading of red blood cells has positive values suggesting a higher work of adhesion compared with that of cohesion and, at the same time, the role of these blood components in blood clotting. Red blood cell deposition in excess is mediated by fibrinogen, for which the spreading work is positive, which means a higher work of adhesion. The positive values determined for fibrinogen spreading work demonstrate its ability to mediate red blood cell adhesion and aggregation by binding to the IIb/IIIa integrin receptor glycoprotein [55]. Thus, fibrinogen is a key protein that influences cascading blood clotting and determines the polymeric material compatibility. Unlike red blood cells, platelets that obtained negative values for the spreading work are associated with a lower work of adhesion compared with that of cohesion. In other words, a good hydrophobicity is correlated with a higher adhesion of the red blood cells on the analyzed surfaces, while the exposure of cellulose acetate/silica substrate to platelets determines an increase in platelets cohesion, with the maximum negative value for a TEOS content of 1 wt.%. The negative values of the spreading work of platelets indicate that the samples do not interact with these blood components, thus preventing activation of coagulation at the blood-biomaterial interface. It is very well known that red blood cells and platelets have an important role in determining the biocompatibility of polymeric materials. Adhesion of the red blood cells at cellulose acetate/silica substrate implies the knowledge of the interactions with blood components. In this context, the two components of the vascular endothelium surface, namely the endothelial glycocalyx and mucopolysaccharides, reject the coagulation factors and platelets that facilitate the appearance of thrombus [56].

In addition to the adsorption properties of fibrinogen on the cellulose/silica surfaces, those of albumin and immunoglobulin (IgG) were also studied. As literature data mention, the adsorption of the plasma proteins occurs in the first phase from the moment of polymeric material exposure to the blood environment. They turn into activated proteins that can catalyze, mediate or moderate the biological response of the biomaterial [57]. There are many studies presented in the literature related to the plasma protein’s adsorption to polymeric surfaces. In some of these was reported that the hydrophobic polymeric surfaces favor the plasma proteins adhesion [58,59], while in others, that the hydrophilic surfaces are the most sensitive to their presence [60,61]. In vitro studies have shown that the albumin has an affinity for hydrophobic surfaces that was justified by its distortion induced by the interaction of the hydrophobic core with the hydrophilic surface [62]. In the present study, from theoretical evaluations, small negative values of albumin and IgG spreading work have resulted. For both plasma proteins this parameter presents the same variation, namely, slightly decrease with the addition of TEOS, reaching a maximum negative value for 1.0 wt.% TEOS, after which a tendency towards positivity appears. Thus, conforming to the theoretical results (Figure 12), it is observed that the proteins bind more firmly to hydrophobic surfaces than to hydrophilic ones. The main reason for this behavior is an increase in the interactions between the hydrophobic polymeric surfaces and hydrophobic domains of the proteins that are exposed during the adsorption process [63]. The small negative values of the albumin spreading work together with the platelet rejection indicate the important role played in the material-host interactions.

The data obtained through these theoretical approximations highlight the biocompatibility of cellulose acetate/silica composite films and can represent the basis information for future in vitro studies concerning their use as bioscaffolds in cell development.

## 4. Conclusions

Cellulose acetate/silica composite films were investigated in order to obtain information on their morphological and topographical aspects, hydrophilic/hydrophobic properties and blood compatibility. The sol-gel technique for CA/TEOS casting solution obtaining, as well as the films’ preparation history, have a major impact on the mentioned properties. The results related to morphological and topographical aspects suggest that:

- composite films containing small amounts of TEOS show nodules of small widths with high heights and the roughness slightly higher than that of the CA sample. For these films the silica nanoparticles are uniformly distributed;
- films with high content of TEOS present nodules whose widths increase and the surface’s roughness reaches the highest values. For these samples, the silica nanoparticles form aggregates non-uniform distributed.

Concerning the wetting properties was found that up to a content of 1.0 wt.% TEOS they are influenced by the surface roughness, after which, the surface chemistry seems to have a greater impact on these features. In this context, it was observed that:

- for all studied samples the disperse components are always higher than the polar ones, while the electron acceptor parameters are smaller than the electron donor parameters;
- for cellulose acetate/silica composite films with low TEOS content the uniform distribution of the silica nanoparticles and roughness characteristic lead to increasing of the samples hydrophobicity;
- for samples with high contents of TEOS, the increase in surface oxygenation, confirmed by XPS analysis, leads to an increase in the sample hydrophilicity.
- values of the surface free energy, interfacial free energy, and work of spreading of water suggest that the samples with low contents of TEOS have a lower wetting capacity compared with those with high contents of TEOS;

According to the theoretical evaluation of the biocompatibility it was shown that:

- work of spreading of red blood cells has positive values that means a higher work of adhesion compared with that of cohesion—this suggesting the role of the red blood cells in blood clotting;
- work of spreading of platelets has negative values that are associated with a lower work of adhesion compared with that of cohesion. The obtained data indicate that the samples do not interact with these blood components, thus preventing the activation of coagulation at the blood-biomaterial interface.
- the fibrinogen spreading work has positive values that suggest his ability to mediate the red blood cells adhesion and aggregation;
- the spreading work of albumin and IgG shows that proteins bind more firmly to the hydrophobic surfaces than to hydrophilic ones. The small negative values of the albumin spreading work and platelets rejection indicate the important role played in the material-host interactions.

Taking into account the results concerning the morphological characteristics but also the wetting and biocompatibility properties can be asserted that the most suitable membranes/films that can be used as bioscaffolds in cell development are those containing up to 1.5 wt.% TEOS.

Consequently, the relationship between the different physical and chemical characteristics of the analyzed membranes surfaces and their biocompatibility properties was established in order to evaluate the cellulose acetate/silica performance in medical applications as biomaterials that modulate extracellular matrix.

## Figures and Tables

**Table 1:** Surface roughness parameters, including the root-mean-square roughness (Sq) and average roughness (Sa) and SiO_2_ nanoparticles sizes.

Sample	Sq (nm)	Sa (nm)	SiO_2_ Nanoparticles Sizes (nm)
CA	7.07	5.48	-
CA+0.5 wt.% TEOS	7.94	5.91	170
CA+1.0 wt.% TEOS	8.32	6.86	110
CA+1.5 wt.% TEOS	22.41	17.22	90
CA+2.0 wt.% TEOS	24.36	18.79	50

**Table 2:** Atomic concentrations at the cellulose acetate/silica films surfaces.

Sample	Element (at %)		
	C (at %)	O (at %)	Si (at %)
CA	61.3	38.7	-
CA+0.5 wt.% TEOS	61.2	38.2	0.6
CA+1.0 wt.% TEOS	57.6	39.4	3.0
CA+1.5 wt.% TEOS	53.5	42.6	3.9
CA+2.0 wt.% TEOS	52.9	43.1	4.0

The obtained data have an error of ±0.1%.

**Table 3:** Contact angle θ (°C), between the different test liquids and cellulose acetate/silica films.

Sample	Water (W)	Diiodmethane (MI)	Ethylene Glycol (EG)
CA	62.81 ± 0.12	40.77 ± 0.33	38.99 ± 0.34
CA+0.5 wt.% TEOS	67.57 ± 0.02	41.31 ± 0.16	41.80 ± 0.05
CA+1.0 wt.% TEOS	76.56 ± 0.25	43.62 ± 0.22	43.27± 0.31
CA+1.5 wt.% TEOS	69.30 ± 0.20	38.56 ± 0.53	38.20 ± 0.55
CA+2.0 wt.% TEOS	59.71 ± 0.37	41.05 ± 0.16	33.79 ± 0.31

**Table 5:** Surface tension parameters ($\mathrm{mN}\cdot m^{-1}$) and contribution of the polar component to the total surface tension for films obtained from CA/TEOS solutions in tetrahydrofuran according to the acid/base method [Equations (1)–(4)].

Sample	Acid/Base Method				
	$\gamma_{\mathrm{lv}}^{d}$	$\gamma_{\mathrm{lv}}^{p}$	$\gamma_{\mathrm{lv}}^{+}$	$\gamma_{\mathrm{lv}}^{-}$	$\gamma_{\mathrm{sv}}^{}$
CA	32.95	7.27	0.69	19.04	40.22
CA+0.5 wt.% TEOS	33.44	6.10	0.64	14.59	39.54
CA+1.0 wt.% TEOS	34.08	4.84	0.91	6.41	38.92
CA+1.5 wt.% TEOS	35.38	6.94	0.82	11.22	42.32
CA+2.0 wt.% TEOS	32.57	9.33	1.02	20.77	41.90

**Table 6:** Surface parameters evaluated based on adsorption/desorption isotherms: water vapor sorption capacity; W, final weight; $r_{pm}$, and average pore size.

Sample	W (%)	$r_{\mathrm{pm}}(\mathrm{nm})$
CA	8.9831	0.75
CA+0.5 wt.% TEOS	14.3958	0.86
CA+1.0 wt.% TEOS	15.1665	0.87
CA+1.5 wt.% TEOS	13.2087	1.03
CA+2.0 wt.% TEOS	9.5118	0.93

Determined based on desorption branch of the isotherm (registered up to a relative humidity of 40%).
